# Comparative Proteomic Analysis of Two Commonly Used Laboratory Yeast Strains: W303 and BY4742

**DOI:** 10.3390/proteomes11040030

**Published:** 2023-10-09

**Authors:** Valentina Rossio, Xinyue Liu, Joao A. Paulo

**Affiliations:** Department of Cell Biology, Harvard Medical School, Boston, MA 02115, USA; xinyue_liu@hms.harvard.edu

**Keywords:** TMTpro, yeast, genetic background, stationary phase, proteome analysis

## Abstract

The yeast *Saccharomyces cerevisiae* is a powerful model system that is often used to expand our understanding of cellular processes and biological functions. Although many genetically well-characterized laboratory strains of *S. cerevisiae* are available, they may have different genetic backgrounds which can confound data interpretation. Here, we report a comparative whole-proteome analysis of two common laboratory yeast background strains, W303 and BY4742, in both exponential and stationary growth phases using isobaric-tag-based mass spectrometry to highlight differences in proteome complexity. We quantified over 4400 proteins, hundreds of which showed differences in abundance between strains and/or growth phases. Moreover, we used proteome-wide protein abundance to profile the mating type of the strains used in the experiment, the auxotrophic markers, and associated metabolic pathways, as well as to investigate differences in particular classes of proteins, such as the pleiotropic drug resistance (PDR) proteins. This study is a valuable resource that offers insight into mechanistic differences between two common yeast background strains and can be used as a guide to select a background that is best suited for addressing a particular biological question.

## 1. Introduction

The budding yeast *Saccharomyces cerevisiae* is a widely used model system for investigating many cellular processes. This unicellular eukaryotic organism has a short life cycle (~90 min), is simple to grow and manipulate, and offers a broad collection of readily available molecular and genomic tools (e.g., yeast deletion and GFP-tagged ORF strain libraries). Because many biochemical and biological pathways are often conserved from yeast to human [1,2], this organism has contributed and continues to contribute to our understanding of molecular mechanisms that are often dysregulated in many human diseases, such as cancer and neurodegenerative disorders.

*S. cerevisiae* laboratory strains are genetically well characterized and can have various genetic backgrounds [3]. Mutations or deletions of some genes can have a different effect depending on the genetic background. For example, Fus3 is required for mating in the SC288 background but not in the W303 background [4], and the deletion of the Igo proteins (Igo1/2) or deletion of the kinase Rim15 severely impairs growth at low temperatures in the W303 background compared to the BY4741 background [5]. Many genes have also been shown to be conditionally essential depending on the genetic background used [3]. Consequently, specific genetic backgrounds are more suitable for studying particular biological processes. For example, the W303 background [6,7] is commonly preferred for cell cycle studies due to the ease of synchronization. Yet, the BY4742 [8] is one of the most used backgrounds as multiple genomic and molecular tools are commercially available (i.e., yeast deletions or ORF-tagged library) in this genetic background.

Both specific genetic mutations and dissimilarities in protein expression contribute to the differences among yeast genetic backgrounds. Thus, characterizing and comparing their proteomes is important to understanding and discerning common and fully background-specific characteristics and/or phenotypes. Until now, the proteomes of the W303 and of the BY4742 genetic backgrounds have not been compared directly side by side. The W303 proteome has been previously compared to other genetic backgrounds, such as CEN.PK2 and FY1679, using mass spectrometry analysis applied to two-dimensional gel electrophoresis [9]. In that study, the proteome was compared during exponential growth, and it was not investigated if the changes observed at the proteome level for different backgrounds were growth-condition-dependent. We emphasize that even though the BY4742 genetic background is commercially available and broadly used by many laboratories, a direct proteome-wide comparison has not been made with the other backgrounds.

We compared the whole proteome abundance profile of W303 and BY4742 genetic backgrounds of *S. cerevisiae* in different growth conditions (exponential and stationary growth phase) using quantitative isobaric-tag-based mass spectrometry techniques. We quantified hundreds of differentially abundant proteins when comparing the two backgrounds during both growth phases. We hypothesize that proteome differences between these two genetic backgrounds could also be influenced by the growth conditions used. Ultimately, this study is an important resource that offers insight into the interpretation of mechanistic differences observed between these two backgrounds. These data can also guide the selection of the proper background that is best suited for a given experiment, and consequently to answer specific biological questions.

## 2. Material and Methods

### 2.1. Materials

The reagents used in this work are commercially available. The *S. cerevisiae* BY4742 strain was purchased from Horizon Scientific (Cambridge, UK), while the W303 strain was a gift from S. Piatti. YPD media was from Sunrise Science (Knoxville, TN, USA). The protease inhibitors and BCA kit used during cell lysate preparation were from ThermoFisher Scientific (Rockford, IL, USA). Trypsin and Lys-C proteases were acquired from ThermoFisher Scientific (Rockford, IL, USA) and Fujifilm Wako (Richmond, VA, USA), respectively. Reagents used for proteomic sample preparation were the following: mass-spectrometry-grade water and organic solvents (J.T. Baker; Center Valley, PA, USA), tandem mass tag (TMTpro) isobaric reagents (ThermoFisher Scientific; Rockford, IL, USA); StageTip Empore-C18 disks were obtained from CDSanalytical (Oxford, PA, USA), while Sep-Pak cartridges (50 mg) were purchased from Waters (Milford, MA, USA).

### 2.2. Yeast Growth and Protein Extraction

*S. cerevisiae* BY4742 and W303 cultures were each grown in parallel overnight in triplicate at 30 °C in YPD medium supplemented with 50 mg/L adenine. The next day, the triplicate cultures were divided into two sets. One set of cultures was diluted with fresh media to OD_600_ = 0.15 and grown until OD_600_ = 0.8 (exponential phase, e). The other set was not diluted with fresh media and was allowed to grow for another 24 h (stationary phase, s) at 30 °C. Cells were collected by centrifugation (2000× *g* for 2 min), washed twice with cold water, flash-frozen in liquid nitrogen, and stored at −80 °C until sample processing. Cell lysis and protein extraction were performed as follows. Briefly, cell pellets were resuspended in lysis buffer (8 M urea, 200 mM EPPS (4-(2-hydroxyethyl)-1-piperazinepropanesulfonic acid), pH 8.5 supplemented with protease inhibitors) and lysed by bead-beating (five cycles of 30 sec with beating alternating on and off) in the cold room. Protein concentrations were determined using a BCA assay performed according to manufacturer’s instructions. Proteins were reduced with 5 mM tris(2-carboxyethyl)phosphine (TCEP) for 20 min, alkylated with 10 mM iodoacetamide for 20 min (in the dark), and finally quenched with 10 mM dithiothreitol (DTT) for 20 min (in the dark). All reactions were incubated at room temperature. A total of 100 µg of protein from each sample was precipitated by chloroform–methanol precipitation.

### 2.3. Protein Digestion, TMT Labeling, and Sample Processing

Samples were digested using Lys-C (overnight at 24 °C) and trypsin (6 h at 37 °C). A total of 1 µg of each enzyme was used per 100 µg of protein. A final volume of 30% acetonitrile was added to each digest followed by the addition of specified tandem mass tag (TMTpro) labeling reagents. A total of 50 µg of peptide for each sample was labeled with 100 µg of the appropriate TMTpro reagent as follows: W303 triplicates in stationary phase: 126, 127n, and 127c; BY4742 triplicates in stationary phase: 128n, 128c, and 129n; W303 triplicates in exponential phase: 129c, 130n, and 130c; BY4742 triplicates in exponential phase: 131, 131c, and 132n. Samples were incubated for one hour at room temperature. Before continuing sample processing, ~1 µg of peptide was collected from each sample, mixed, and desalted via StageTip [10] to verify labeling efficiency (ensuring that it was > 97%). Hydroxylamine (final concentration of ~0.3%) was added to each sample to quench the labeling reaction. Samples were incubated at room temperature for 15 min. Then, samples were pooled 1:1 and desalted using a 50 mg Sep-Pak solid-phase extraction column. Fractionation was executed with a basic pH reversed-phase (BPRP) HPLC. An Agilent 1200 pump (Lexington, MA, USA) with an Agilent 300 Extend C18 column (3.5 μm particles, 2.1 mm ID, and 250 mm in length) was used. Peptides were fractionated by applying a 50 min gradient that is linear from 5% to 35% acetonitrile in 10 mM ammonium bicarbonate pH 8 and at a flow rate of 0.25 mL/min. We collected 96 fractions that we concatenated and condensed down to 24 superfractions, as described elsewhere [11]. We obtained two sets of 12 non-adjacent superfractions from these 24 superfractions. We acidified the superfractions with formic acid to a concentration of 1% followed by vacuum centrifugation. Each superfraction was desalted via StageTip, dried again by vacuum centrifugation, and reconstituted in 5% acetonitrile, 5% formic acid.

### 2.4. Mass Spectrometry Data Acquisition and Processing

Mass spectrometric data were acquired on an Orbitrap Fusion Lumos mass spectrometer which was in line with a Proxeon NanoLC-1200 UHPLC and a FAIMSpro gas-phase fractionation interface [12]. A 100 μm capillary column was manufactured in-lab and packed with 35 cm of C18 beads (Accucore150, 2.6 μm, 150 Å; ThermoFisher Scientific). Data were acquired over a 90 min gradient. The scan sequence began with an MS1 spectrum that was acquired in the Orbitrap (resolution was 60,000, scan range was 350–1350 Th, automatic gain control (AGC) target was set as “standard,” and the maximum injection time was set to auto. The RTS-MS3 scan sequence method was used to reduce ion interference [13,14]. MS2 analysis consisted of collision-induced dissociation (CID) and ion trap analysis (automatic gain control (AGC) was 2 × 10^4^, maximum injection time was 35 ms, q-value was 0.25, normalized collision energy (NCE) was set at 35%, and the isolation window was set at 0.7 Th). We used the Real Time Search (RTS) option with an *S. cerevisiae* yeast database (UniProt, downloaded August 2021), and we limited MS3 scans to 2 peptides per protein per fraction. After acquiring a matched MS2 spectrum, we acquired an MS3 spectrum in which multiple MS2 fragment ions were captured using an isolation waveform with multiple frequency notches. MS3 precursors were fragmented by higher-energy collisional dissociation (HCD) and analyzed using the Orbitrap (NCE was 55%, AGC was set at 1.5 × 10^5^, maximum injection time was 150 ms, and the resolution was 50,000, which was sufficient to discriminate between TMT isotopologues). In all, 24 RAW files were acquired. For one set of 12 non-adjacent superfractions, we used a FAIMS compensation voltage (CV) set of −40/−60/−80 V, while for the other 12 superfractions, we used a CV set of −30/−50/−70 V. A 1.25 sTopSpeed cycle was used for each CV.

Spectra were converted to mzXML via MSconvert [15], after which database searching included all *S. cerevisiae* entries from UniProt (the same database as used for RTS, above) and all protein sequences in that database in reverse order. Searches were performed using a 50 ppm precursor ion tolerance and a product ion tolerance of 0.9 Da to maximize sensitivity in conjunction with Comet database searching and linear discriminant analysis (LDA) [16,17]. TMT tags on lysines and peptide N-termini (+304.207 Da) and carbamidomethylation of cysteines (+57.021 Da) were set as static, whereas oxidation of methionines (+15.995 Da) was set as a variable modification. Peptide-spectrum matches (PSMs) were adjusted to a 1% false discovery rate (FDR) [18,19], and filtering thereof was performed using LDA [19] to assemble the dataset further to achieve a final protein-level FDR of 1% [20]. Once completed, proteins were quantified by summing reporter ion counts across matching PSMs [21]. Reporter ion intensities were adjusted for the isotopic impurities of the TMT reagents as specified by the manufacturer. The signal-to-noise (S/N) measurements of peptides assigned to each protein were summed and normalized such that the sum of the signal for all proteins in each channel was the same, thereby accounting for equal protein loading (i.e., column normalization). Finally, each protein abundance measurement was represented as a percentage of the total, in that the summed S/N for that protein across all channels was 100, thus providing a relative abundance (RA) measurement. We determined protein abundance alterations to be statistically significant if meeting a fold change cutoff |log_2_ ratio| >1 and an uncorrected *p*-value of less than 0.01.

## 3. Results and Discussion

### 3.1. Whole Proteome Abundance Profiling Revealed Hundreds of Proteins That Differed in Abundance between BY4742 and W303 Yeast Genetic Backgrounds 

**Figure 1:**
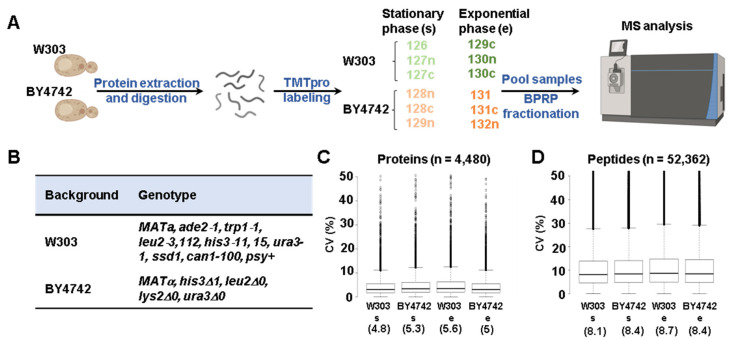
Experimental workflow, genotypes of the strains used, and coefficient of variation among replicates. (**A**) Wild-type yeast cells of W303 and BY4742 genetic backgrounds were grown in exponential (e) and stationary (s) phase in triplicate. Cells were harvested and lysed, after which proteins were precipitated. Following digestion with LysC and trypsin, the peptides were labeled with tandem mass tag (TMTpro) reagents, as indicated, pooled 1:1, and fractionated by basic pH reversed-phase (BPRP) HPLC. This panel has been assembled, in part, using Biorender.com. (**B**) Genotypes of the *S. cerevisiae* strains used in the experiment. Distribution of the coefficient of variation (CV) for the replicate measurements at both the (**C**) protein and (**D**) peptide level. The median CV is indicated below the graphs.

We used isobaric-tag-based quantitative profiling (Figure 1A) to compare the proteomes of two *S. cerevisiae* genetic backgrounds, BY4742 and W303 (Figure 1B), in different growth phases (exponential and stationary). In total, we matched 52,362 non-redundant (unique) peptides belonging to 4480 proteins that we quantified across all the conditions in both genetic backgrounds. All proteins, peptides, and their associated relative abundances used for quantitation were reported in Tables S1 and S2.

**Figure 2:**
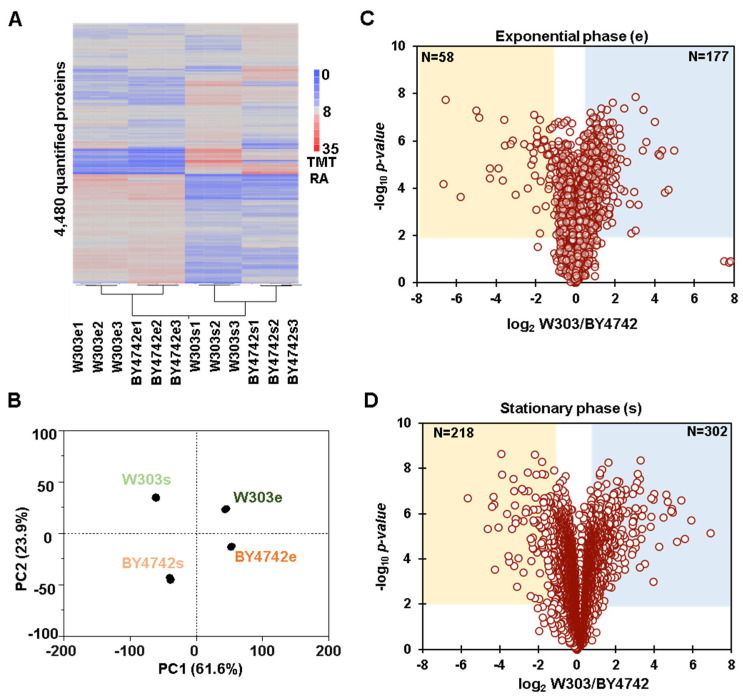
Proteome-wide abundance profiling of differentially abundant proteins when comparing the two yeast genetic backgrounds. (**A**) Hierarchical clustering analysis of the TMT relative abundance (TMT RA) for proteins quantified across the 12 TMT channels. (**B**) Principal component analysis (PCA) of the dataset reflects the clustering of the replicates (**C**,**D**). The volcano plots illustrate differentially abundant proteins (i.e., |log_2_ ratio| >1 and *p*-value < 0.01) between the two yeast genetic backgrounds in both (**C**) exponential and (**D**) stationary growth phases.

We first explored the global protein abundance differences between the two yeast genetic backgrounds. We calculated the coefficient of variation (CV) of each condition using the average of three replicates to assess the quantitative reproducibility of the experiment. The medians of the CV distribution for both proteins and peptides were similar across the conditions tested and were less than 6% and 9%, respectively (Figure 1C,D). We performed hierarchical clustering using Euclidean distance with Ward’s inter-cluster linkage using the values of the 4480 proteins that were first scaled to 100 across the twelve channels of the experiment (Figure 2A). The samples clustered as expected, as the triplicates of each genetic background and growth condition (exponential and stationary phase) clustered together. Similarly, principal component analysis (PCA) of the dataset confirmed the tight clustering of the replicates in all conditions tested (Figure 2B). In fact, the first two principal components (PC1 and PC2) together accounted for ~85% of the variance. PC1 (presumably growth phase) explained more than 60% of the variance, while PC2 (presumably background) explained 24% of the variance (Figure 2B). Overall, we observed considerable differences in the whole proteome with respect to both the genetic background and the growth phase. We found 235 differentially abundant proteins (±2-fold change, *p*-value < 0.01) with respect to the genetic background during the exponential growth phase. In particular, 177 proteins were significantly more abundant in the W303 background, while 58 were so in the BY4742 background (Figure 2C). The abundances of the remaining 4245 proteins, which constituted nearly 95% of the quantified proteins, did not change significantly. We determined a greater number of proteins to be differently abundant when comparing the two backgrounds during the stationary phase. In fact, 302 proteins were significantly more abundant in the W303 background and 218 in the BY4742 background, while 3960 did not change (representing nearly 81% of the quantified proteins) (Figure 2D).

As an example, we highlighted the pleiotropic drug resistance (PDR) protein family [21]. This class of proteins determines the sensitivity or resistance of yeast cells to drugs or small molecules present in the environment (i.e., the media). We found that several proteins in this family were differentially abundant with respect to genetic background and growth phase (Figure S1). In total, we detected seven PDR proteins in our experiment. Only the protein abundance of Pdr1, a transcription factor, was not considerably altered (Figure S1A), whereas the remaining PDR protein profiles did change across conditions. For example, the abundance of the plasma membrane transporter Pdr5 was higher in the BY4742 background compared to the W303 background during exponential growth (Figure S1B). Also, the abundances of both the membrane transporter Pdr10 and the phosphatidylinositol transfer protein Pdr16 were lower in the BY4742 background when in the stationary phase (Figure S1C,F), while the plasma membrane transporter Pdr12 and the phosphatidylinositol transfer protein Pdr17 were both higher in the BY4742 background again when in the stationary phase (Figure S1D,G). Lastly, the abundance of the plasma membrane transporter Pdr15 was higher in the BY4742 background in both exponential and stationary growth phases (Figure S1E). The varying expression of PDR proteins could contribute to the different drug sensitivity observed for these two strains, as reported previously [22]. In summary, our proteome profiling experiment revealed that the abundance of several hundred proteins was significantly altered in these two genetic backgrounds and suggested that the degree of divergence in protein expression was largely growth-phase-dependent.

### 3.2. Proteomic Analysis Confirms the Mating Type of the Strains Used in This Experiment 

Haploid *S. cerevisiae* cells exist as two different mating types, a and alpha (a). To become diploid, yeast cells of mating type *a* (*MATa*) secrete a specific molecule, mating factor a, which attracts them to the cells of mating type alpha (*MATα*) and vice versa. The mating type factors bind specific receptors on the membrane of the opposite mating type cells [23]. Usually, the mating type of research laboratory yeast strains is known, but in some cases, the mating type must be determined (such as after tetrad dissections) or confirmed (such as after purchasing the strain from a commercial vendor or when receiving it as a gift from another laboratory). Thus, we analyzed the abundance profiles of mating-type-specific proteins detected in our experiment to verify the mating type of our two yeast strains.

**Figure 3:**
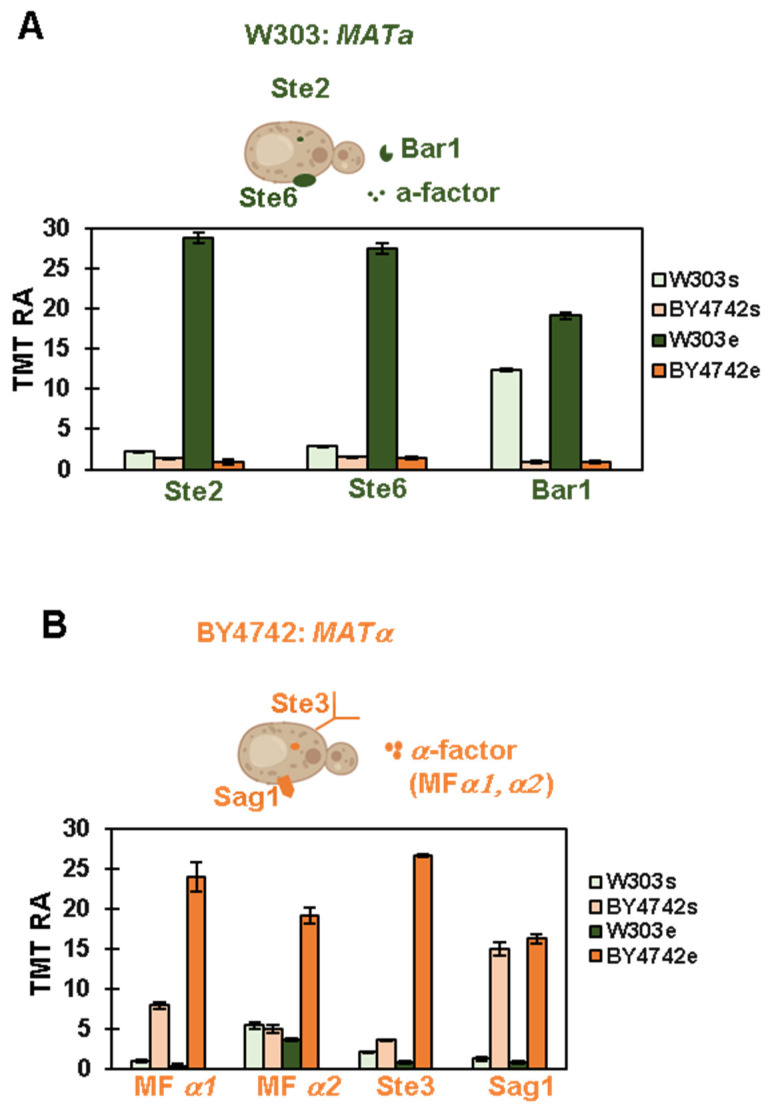
Analysis of mating-type-specific proteins. The TMT RA (relative abundance) values are shown for proteins involved in the mating type pheromone signaling pathway. (**A**) The highlighted proteins are specific for mating type a (W303): the alpha-factor receptor (Ste2), the a-factor transporter required to export a-factor (Ste6), and the protease Bar1. (**B**) The highlighted proteins are specific for mating type alpha (BY4742): the mating factor α: MFα1 and MFα2; the a-factor receptor (Ste3); the alpha cells-specific agglutinin protein (Sag1). This figure has been created, in part, using Biorender.com. Error bars represent the standard deviation of triplicate measurements.

In the case of the W303 background, we detected three proteins specific to *MATa* cells, confirming that these cells are indeed of mating type a (Figure 3A). Specifically, we matched Ste2, which is the alpha-factor receptor; Ste6, which is required for the export of the a-factor outside the cells; and the protease Bar1, which is secreted only by *MATa* cells to inactivate the alpha-factor (Figure 3A). Interestingly, the relative abundance of these proteins was very low in stationary phase apart from Bar1 (Figure 3A). To be specific, the abundance of Ste2 and Ste6 was approximately 10 times higher in exponential phase than in stationary phase. In BY4742 cells, only a negligible amount of these proteins was detected, suggesting that the BY4742 cells were of the opposite mating type (*MATα*). Consistent with these results, we quantified four proteins specific to *MATα* cells in the BY4742 background, including the mating factor alpha itself (MFα1 and MFα2), the a-factor receptor (Ste3), and the *MATα* cell-specific agglutinin (Sag1). These proteins, except for Sag1, were much higher in abundance in the exponential growth phase compared to the stationary growth phase. Specifically, MFα1, MFα2, and Ste3 were respectively three, four, and seven times lower (Figure 3B). These data suggested that yeast cells in the stationary phase could be unable to mate efficiently, if at all. Thus, isobaric-tag-based quantitative proteomic analysis could be used successfully for mating type confirmation or determination. We anticipate further development and refinement of such an assay to be developed in a high-throughput and/or targeted format [24].

### 3.3. Protein Profiles of Auxotrophic Markers and Their Metabolic Pathways

**Figure 4:**
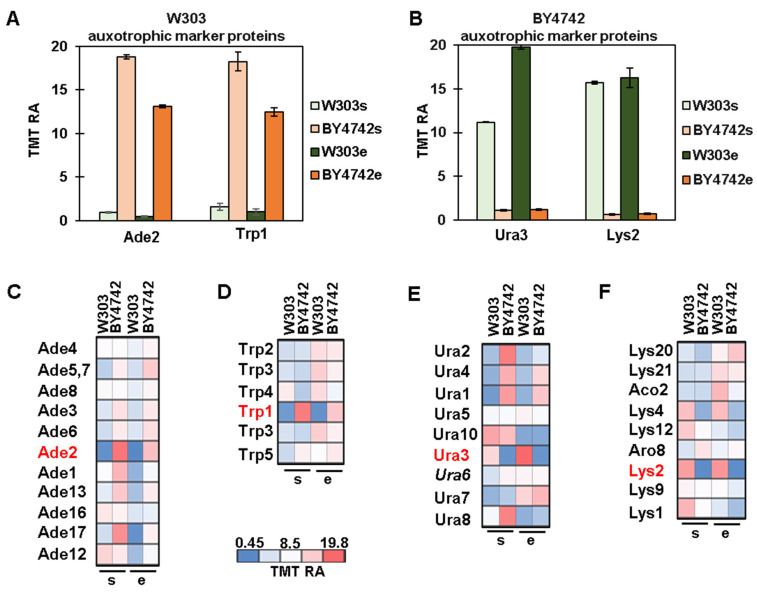
Analysis of background-specific auxotrophic marker proteins and their associated metabolic pathways. TMT relative abundance (TMT RA) of auxotrophic marker proteins for (**A**) W303-specific or (**B**) B4742-specific strains. Error bars represent the standard deviation of triplicate measurements. TMT RA of the metabolic enzymes of the indicated pathways, specifically (**C**) adenine, (**D**) tryptophan, (**E**) uracil, and (**F**) lysine. Metabolic enzymes corresponding to auxotrophic markers are shown in red.

Yeast strains routinely used in research laboratories are auxotrophic and, as such, have mutations/deletions in metabolic genes, such as *URA3* or *HIS3*, that can be used to select cells that have been transformed with exogenous DNA carrying a wild-type copy of these genes [25]. Usually, each genetic background has its own specific auxotrophic markers [3]. The W303 background, for example, carries the following auxotrophic mutations (genotypes listed in Figure 1B): *trp1-1*, *ade2-1*, *ura3-1*, *his3-11,15*, and *leu2-3112* [3]. The BY4742 background instead has the following genes deleted: *HIS3*, *URA3*, *LYS2,* and *LEU2* [3]. Consequently, we quantified the proteins encoded by these genes to confirm the background of the strains we used in the experiment. Mutations in the *TRP1* and *ADE2* genes in the W303 background generated truncated proteins that were likely degraded. In fact, the abundance of Trp1 and Ade2 were, respectively, 30 and 13 times lower in the W303 background (Figure 4A). We also observed a negligible amount of Ura3 and Lys2 specifically in the BY4742 cells (Figure 4B). In particular, Ura3 and Lys2 were, respectively, 16 and 23 times lower in the BY4742 background. This finding is consistent as the BY4742 background carries the deletion of both these genes (Figure 1B). The W303 background carries a point mutation in the *URA3* gene resulting in a single amino acid residue substitution (G701E). This mutation affects the enzymatic activity of Ura3 (and thus, W303 cells do not grow in medium lacking uracil), but the abundance of the protein remained high compared to the BY4742 background (in which the *URA3* gene was deleted). We could not detect Leu2 and His3 in the experiment likely due to little or no expression in either background. Thus, our proteomic analysis was consistent with the expected results regarding the auxotrophic markers in these backgrounds (Figure 1B).

Most auxotrophic markers are key members of metabolic pathways. Perturbation at a single step of a metabolic pathway will likely affect enzymes (either directly or indirectly) in the same pathway, as they adjust their activity in response to such perturbation. Change in protein abundance could lead to alterations of enzymatic activity. Thus, we analyzed the effect of the auxotrophic mutations on the abundance of enzymes in the metabolic pathways associated with these markers. We focused on enzymes from the adenine biosynthetic pathway, for which we observed a strong decrease in Ade2 in W303 cells, in agreement with their genotype (*ade2-1*) (Figure 4C). We also noted a W303-specific decrease in Ade17 (Figure 4C) that was likely a consequence of the reduced activity of Ade2. In the tryptophan biosynthetic pathway, Trp1 was the only enzyme downregulated specifically in W303 cells, which again was consistent with their genotype (*trp1-1*) (Figure 4D). By examining the enzymes involved in uracil biosynthesis, we observed a strong decrease in abundance of Ura3 in the BY4742 background, as reflected in its genotype (*ura3D*). In addition to Ura3, the abundance of Ura4 and Ura1 increased in BY4742 cells, likely as a compensation mechanism for the decrease in Ura3. However, Ura2 increased only in BY4742 cells in the stationary phase (Figure 4E), hence implying a different compensatory mechanism. In the lysine biosynthetic pathway, the abundance of Lys2 was dramatically lower in BY4742 cells, and as such was consistent with its genotype (*lys2Δ*). Likewise, Lys4 was lower in BY4742 cells likely to compensate for Lys2 being deleted. Thus, these data supported the idea that background-specific auxotrophic mutations can alter the protein abundance of other enzymes in their metabolic pathway.

### 3.4. Proteome-Wide Abundance Profiling of the Transition from Exponential to Stationary Growth Phase

**Figure 5:**
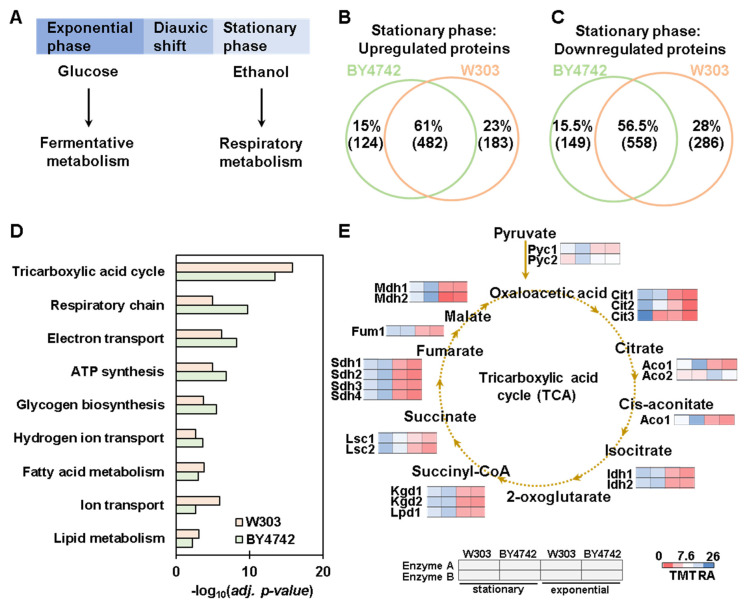
Proteome-wide abundance profiling of the changes induced by the transition from the exponential to the stationary growth phase. (**A**) A diagram of different carbon source metabolism happening during the exponential and stationary phases is shown. Also shown is the overlap between the stationary-phase-induced proteins with statistically significant (**B**) increase and (**C**) decrease in abundance in the two backgrounds. (**D**) Biological processes enrichment analysis of the proteins that are significantly higher in abundance (adjusted *p*-value < 0.01) in the stationary phase. (**E**) Relative abundance profiles of the tricarboxylic acid cycle (TCA) enzymes.

The proteins differentially regulated between the two backgrounds are likely influenced by the growth conditions. As proof of concept, we compared the proteome of these two backgrounds in the stationary phase. The diauxic shift is the transition from the exponential to stationary growth phase and occurs when glucose is exhausted in the medium and yeast cells switch from a glucose-dependent fermentative metabolism to a respiratory metabolism (Figure 5A).

First, we validated our experiment by assessing the abundance of proteins known to be upregulated in the stationary growth phase. We observed an increase in two stationary phase gene (SPG) proteins, Spg1 and Spg4 (Figure S2A), in both backgrounds. Moreover, we confirmed the increase in abundance (in both backgrounds) of Acs1, Fbp1, Agp2, and Hsp26 (Figure S2A) that were reported previously as being upregulated in the stationary phase [26,27]. Next, we analyzed the changes in the proteome induced by the shift to the stationary phase. We observed a higher number of differentially abundant proteins in the BY4742 background compared to W303. In fact, the abundances of 1295 proteins were significantly altered (697 decreased, while 598 increased) in the BY4742 background (Figure S2B). However, only 521 proteins changed significantly (218 decreased, while 303 increased) in the W303 background (Figure S2C). Thus, whereas nearly 90% of the proteome was unaltered in the W303 background, only 74% was so in the BY4742 background.

Next, we compared the differentially abundant proteins in the stationary-to-exponential phase of both genetic backgrounds (Figure 5B). The data revealed that 482 proteins increased in both backgrounds (61%), while 124 were increased specifically in the BY4742 background and 183 in the W303 background. Of the proteins with decreased abundance in the stationary versus exponential growth phase, 558 (56.5%) were common between backgrounds, while 149 decreased specifically in the BY4742 cells and 286 in the W303 cells (Figure 5C). These data suggested that even if most of these proteins were in common, specific proteins were altered in one background but not in the other. We then performed gene ontology analysis using the Database for Annotation, Visualization, and Integrated Discovery (DAVID) [28] to determine which biological processes were over-represented (Benjamini–Hochberg corrected *p*-value < 0.01) by the subsets of proteins that were significantly upregulated in the stationary phase in both the backgrounds. These proteins were enriched for GO terms related to respiratory metabolism, such as tricarboxylic acid cycle, respiratory chain, and electron transport (Figure 5D). This enrichment was consistent with the metabolic changes (from fermentative to respiratory metabolism) that were triggered during the switch from exponential to stationary growth phase. We analyzed in detail the abundance of the enzymes of the tricarboxylic acid (TCA) cycle, which is very active during respiration. The TCA enzymes quantified in our experiment (Figure 5E) included the three citrate synthases (Cit1-3), the two aconitate hydratases (Aco1, Aco2), the two isocitrate dehydrogenases (Idh1, Idh2), the three enzymes of the 2-ketocluterate dehydrogenase complex (Kgd1, Kgd2, Lpd1), the two subunits of the succinyl-CoA ligase (Lsc1, Lsc2), the four subunits of the succinate dehydrogenases (Sdh1, Sdh2, Sdh3 and Sdh4), the fumarate hydrolase (Fum1), and the two malate dehydrogenases (Mdh1, Mdh2). In addition, we quantified the two pyruvate carboxylases, Pkc1 and Pkc2, that convert pyruvate to oxaloacetate, the first metabolite of the TCA cycle (Figure 5E). Most of these enzymes and specifically the major isoforms (detected with a greater number of peptides) were of higher abundance at the protein level in both backgrounds after the transition from the exponential to the stationary phase (Figure 5E). The few exceptions were minor isoforms, such as Pyc2, Cit3, and Aco2. Pyc2 abundance was higher in the exponential compared to the stationary growth phase only in the W303 background; Cyt3 abundance did not change in BY4742 background, and the abundance of Aco2 increased in the exponential phase for both backgrounds (Figure 5E). Thus, although many of the changes at the proteome level that were related to the shift from the exponential to the stationary growth phase were identical between the two backgrounds, some differences were observed, such as the minor isoforms of enzymes of the TCA cycle as described above.

## 4. Conclusions and Limitations

Here, we presented the first quantitative whole-proteome analysis of two commonly used laboratory *S. cerevisiae* strains: W303 and BY4742. We profiled over 4400 proteins that constitute approximately 90% of the yeast proteome. We assigned hundreds of spectra corresponding to peptides which we suspect are differentially regulated between these two backgrounds during the exponential and stationary growth phases. These background-dependent protein abundance alterations can help clarify why some gene mutations and deletions have background-specific phenotypes. Here, we showed also that isobaric-tag-based quantitative proteomic analysis can be used successfully to determine or confirm both the auxotrophic markers specific to a certain genetic background and the mating type, which may be developed as a targeted assay in the future [24]. Our study highlighted growth-phase-dependent differences in protein abundance with respect to yeast background. Indeed, we anticipate that proteins in addition to those that we showcased herein could become differently regulated if the cells were grown under perturbations that we did not test (e.g., altered carbon or nitrogen source, increased or decreased growth temperature, or drug treatments). Here, we have provided a list of proteins that may be altered between two commonly used laboratory yeast strains. This list will serve as a starting point for those who are interested in such mechanisms, or they can be used to support other previously collected data. As such, orthogonal and more targeted experiments will enable a more accurate and comprehensive quantification of specific proteins. We stress the need for orthogonal validation, and potentially manual validation of spectra, for certain proteins of interest as false spectral assignments can be made for data of low spectral quality.

A limitation of our study is that our analysis is based on the consensus protein sequences, indeed proteoforms are not considered as they could not be discriminated in our workflow. Similarly, in our study, we did not consider the most common post-translational modifications (e.g., phosphorylation, acetylation, and ubiquitylation) that could be different between these two backgrounds. This analysis could reveal other differences between these strains. We have created a website with R shiny to allow researchers to easily access our dataset of 4480 proteins and search for the abundance profiles of their proteins of interest: https://wren.hms.harvard.edu/yeastback (accessed on 26 September 2023). The website includes additional tabs that highlight the String network [29] associated with the selected proteins, as well as proteins with a similar expression pattern as determined by Euclidean distance [30] to enable the further extraction of biologically relevant data. In summary, our dataset is indeed a valuable resource that may be accessed to select a yeast strain with a specific background that is more suitable to study a particular biological process.

## Data Availability

The data presented in this study are openly available at the Proteo meXchange Consortium via the PRIDE [31] partner repository with the dataset identifier PXD044388. Reviewer account details: Username: reviewer_pxd044388@ebi.ac.uk Password: dR0V85Jc.

## Supplementary Materials

The following supporting information can be downloaded at: https://www.mdpi.com/article/10.3390/proteomes11040030/s1, Figure S1: Examples of differentially abundant proteins: PDR proteins. Figure S2: Differentially abundant proteins when comparing stationary and exponential growth phase. Table S1: List of the proteins and their associated relative abundances. Table S2: List of the peptides and their associated relative abundances.
