# Supplementary material for: Comparative Proteomic Analysis of Two Commonly Used Laboratory Yeast Strains: W303 and BY4742
Source: Proteomes. 2023 Oct 9;11(4):30. doi: 10.3390/proteomes11040030 (PMC10594481; doi:10.3390/proteomes11040030)
Supplement: Supplementary file 1 [file proteomes-11-00030-s001.zip › Supplementary Figure.pdf]

Figure S1:

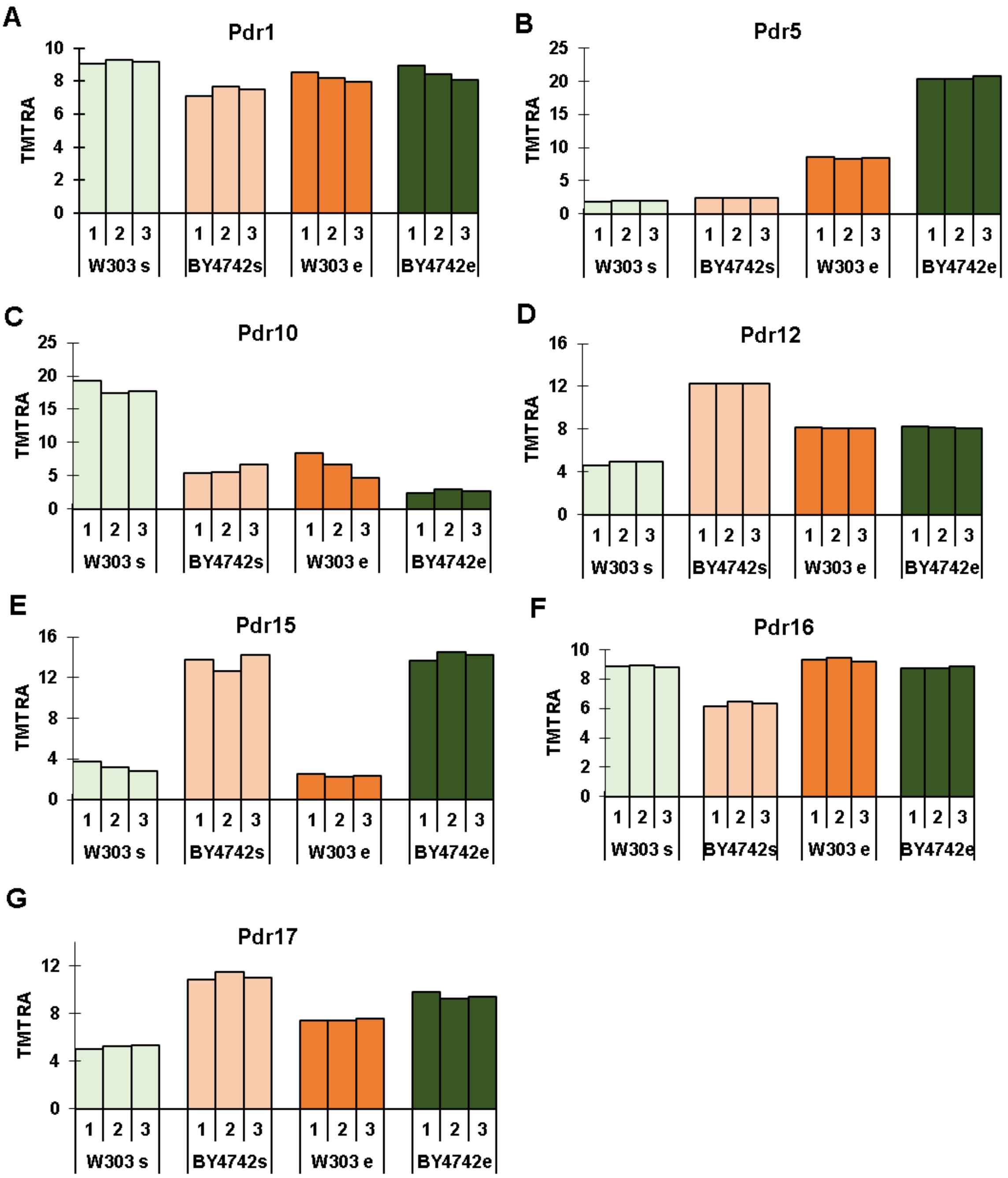

Fig. S1 Examples of differentially abundant proteins: PDR proteins. The TMT relative abundance (RA) is presented for several PDR proteins, which are involved in multidrug resistance. Specifically, we highlight **A)** Pdr1, **B)** Pdr5, **C)** Pdr10, **D)** Pdr12, **E)** Pdr15, **F)** Pdr16, and **G)** Pdr17.

Figure S2:

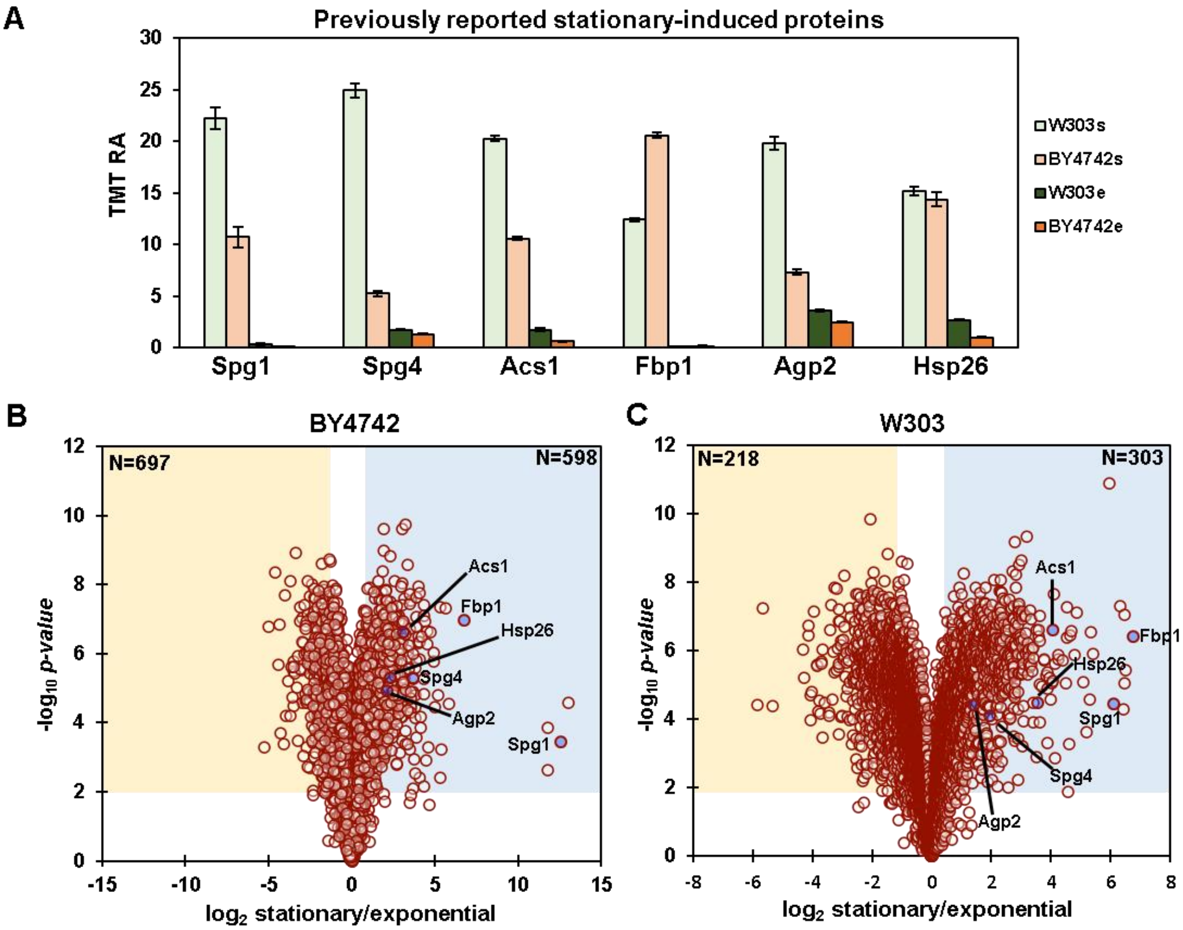

Fig. S2 Differentially abundant proteins when comparing stationary and exponential growth phase. A) The TMT relative abundance (RA) is shown for previously reported stationary growth phase-induced proteins: the SPG proteins (Spg1 and Spg4); the acetyl-coenzyme A synthetase 1, Acs1; the fructose-1,6-biphosphatase, Fbp1; the general amino acid permease, Agp2 and the heat shock protein, Hsp26. The volcano plots illustrate the differentially abundant proteins (*i.e.*, $\log_2 > 1$ or $\log_2 < -1$, *p-value* < 0.01) when comparing the stationary and exponential growth phases in both backgrounds, **B)** BY4742 and **C)** W303. Proteins shown in **A**) are highlighted in the volcano plots.
